# Supplementary material for: Identification of photoperiod-induced specific miRNAs in the adrenal glands of Sunite sheep (Ovis aries)
Source: Front Vet Sci. 2022 Jul 22;9:888207. doi: 10.3389/fvets.2022.888207 (PMC9354845; doi:10.3389/fvets.2022.888207)
Supplement: Supplementary file 1 [file Data_Sheet_1.ZIP › Supplementary file/miRDeep2 file.zip!/miRDeep2 file/Novel_Structure/SPLP42A1/Novel_353.pdf]

Provisional ID	:	NC_019463.2_12080
Score total	:	0.5
Score for star read(s)	:	-1.3
Score for read counts	:	0
Score for mfe	:	0.2
Score for randfold	:	1.6
Score for cons. seed	:	
Total read count	:	13
Mature read count	:	13
Loop read count	:	0
Star read count	:	0

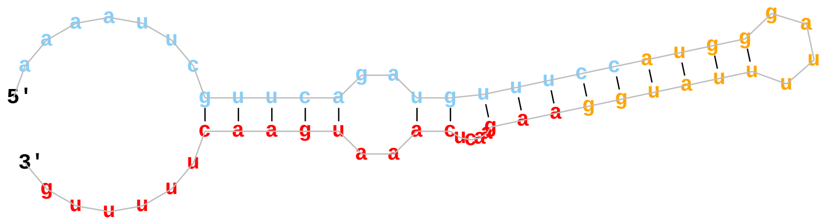

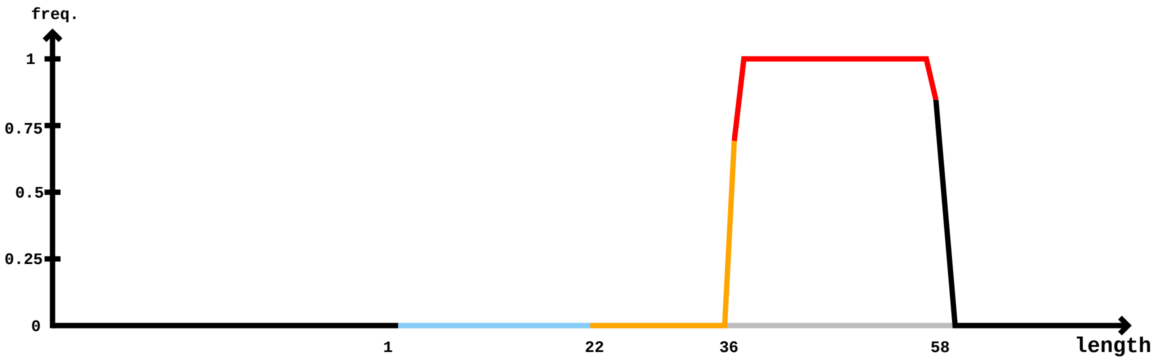


```
                                                      Star                                 Mature
5'- gacagugguaaaaagcuauauacugggcuggccaaaaauucguucagauguuuccaugggauuuuauggaagaacucaaaugaacuuuuuggucaauucaauauuugcuu   -3'    exp
    ............((((...(((.((((.((((((((((((((((......(((((((((....)))))))))......)))))).)))))))))).)))).)))..))))    reads       mm          sample
    .....................................................................aaAaacucaaaugaacuuuuu....................    1           1           seq
    .....................................................................aaAaacucaaaugaacuuuuug...................    8           1           seq
    ......................................................................agaacCcaaaugaacuuuuu....................    1           1           seq
    ......................................................................aAaacucaaaugaacuuuuug...................    3           1           seq
```
